# ‘Positive results of an intensive, immersive, confrontational and protocolized 10-week residential program for youth with mental health problems.’

**DOI:** 10.1192/j.eurpsy.2023.474

**Published:** 2023-07-19

**Authors:** J. Vangeneugden

**Affiliations:** Yes We Can Clinics, Hilvarenbeek, Netherlands

## Abstract

**Introduction:**

Treating mental health problems adequately is of paramount importance given the tremendous burden it places on the individual and on society. Knowing and realizing there is no one size-fits-all-solution, some methods do yield better results. The yardstick as such can be interpreted with scores on questionnaires, subjective accounts and/or having the need for further future follow-up treatments.

**Objectives:**

Within the Yes We Can Clinics, based in the Netherlands, we provide a very intensive 10-week residential treatment program where clients learn to acknowledge their problems and get to the root of these problems. The program is centered around confrontation in group sessions from counselors and peers, a well-thought out activity program from early morning till late evening, multiple psychotherapeutic sessions on a daily basis and if possible, minimize the use of medication.

**Methods:**

Different Routine Outcome Measurement tests were applied.

**Results:**

Here we measured willingness to participate in the program, which fluctuates from average to low at the start, reaching a significant low motivational point after 2/3 weeks, in accordance with the quintessential confrontational aspect, but following the principles of the program in combination with reconnecting with parents and family (systems-approach), a tremendous increase in willingness and commitment towards the program, but also towards life and facing mental health struggles in general, arises.

**Conclusions:**

Herewith confirming the effectiveness of our intensive, immersive, confrontational and protocolized 10-week residential program for youth with mental health problems

**Disclosure of Interest:**

None Declared

